# Association Between C-Reactive Protein–Triglyceride Glucose Index and Depressive Symptoms Among US Adults: A Nationally Representative Cross-Sectional Study From 2005 to 2023

**DOI:** 10.62641/aep.v54i2.2163

**Published:** 2026-04-15

**Authors:** Dan Liu, Wenye Qi, Chunying Zhu

**Affiliations:** ^1^Department of Clinical Psychology, The First Hospital of Jiaxing (Affiliated Hospital of Jiaxing University), 314000 Jiaxing, Zhejiang, China; ^2^Department of Psychiatry, The Third People’s Hospital of Deqing County, 313201 Huzhou, Zhejiang, China; ^3^School of Clinical Medicine, Affiliated Hospital of Hangzhou Normal University, 310036 Hangzhou, Zhejiang, China; ^4^Department of Clinical Psychology, Affiliated Hospital of Hangzhou Normal University, 310036 Hangzhou, Zhejiang, China

**Keywords:** C-reactive protein, triglyceride glucose index, depression, inflammation, insulin resistance

## Abstract

**Background/Objective:**

Depressive disorders represent a major global health challenge, with inflammation and insulin resistance identified as key pathophysiological factors. The C-reactive protein–triglyceride glucose index (CTI), a novel composite biomarker integrating the inflammatory and metabolic pathways, has demonstrated enhanced predictive value in cardiometabolic diseases. However, its relationship with depression remains unexplored. This study examined the association between CTI and depressive symptoms in a nationally representative U.S. adult population.

**Methods:**

We conducted a cross-sectional analysis using National Health and Nutrition Examination Survey data from 2005 to 2023. Depressive symptoms were assessed using the Patient Health Questionnaire-9, with scores ≥10 indicating clinically significant symptoms. CTI was calculated as 0.412 × Ln(CRP) + Ln[triglycerides (mg/dL) × fasting glucose (mg/dL)/2]. Multivariable logistic regression models were employed to evaluate CTI–depression associations, adjusting for sociodemographic factors, comorbidities and laboratory parameters. Restricted cubic spline analysis assessed dose–response relationships, and subgroup analyses examined consistency across demographic and clinical strata.

**Results:**

Among 15,318 participants (mean age 48.97 years; 49.78% female), 8.73% exhibited depressive symptoms. After comprehensive adjustment, each unit increase in CTI corresponded to a 23% increase in the risks of depression (odds ratio (OR) = 1.23, 95% confidence interval (CI): 1.11–1.36, *p* = 0.0001). Participants in the highest CTI tertile demonstrated 48% elevated odds compared with those in the lowest tertile (OR = 1.48, 95% CI: 1.17–1.86, *p* = 0.0009), with a significant linear trend (*p* for trend = 0.0005). Restricted cubic spline analysis confirmed a linear dose–response relationship (*p* for nonlinearity = 0.1665). Associations remained consistent across age, sex, race/ethnicity and comorbidity subgroups (all *p* for interaction >0.05).

**Conclusion:**

Elevated CTI levels are independently associated with increased depression risk in U.S. adults, demonstrating a linear dose–response relationship. CTI may serve as a practical screening tool for identifying individuals at heightened depression risk, enabling integrated cardiometabolic–mental health interventions.

## Introduction

Depressive disorders represent a major healthcare challenge, affecting roughly 4.4% of individuals worldwide [1]. The prevalence of depressive symptoms has increased substantially over recent decades, with studies indicating a nearly 50% rise in reported cases between 1990 and 2017 [2]. The pathogenesis of depressive disorders involves multifaceted interactions among genetic predisposition, environmental exposures and physiological mechanisms. Within this complex framework, inflammatory processes and metabolic dysregulation have gained recognition as important pathophysiological contributors. Insulin resistance is particularly noteworthy. Accumulating research indicates that these metabolic and inflammatory pathways are integral to depression onset and persistence [3, 4]. Evidence from diverse studies revealed that individuals with depressive presentations exhibit increased inflammatory markers, with C-reactive protein (CRP) being particularly prominent [5, 6]. Meanwhile, insulin resistance has emerged as a distinct risk factor contributing to depression onset [7, 8].

Recent research has focused on identifying novel biomarkers that integrate multiple pathophysiological pathways involved in depression. The TyG index, derived from fasting triglyceride and glucose measurements, has gained recognition as a dependable proxy indicator for insulin resistance [9, 10]. Similarly, CRP serves as a well-validated biomarker reflecting systemic inflammatory status [11]. However, these isolated biomarkers may inadequately reflect the intricate interactions between metabolic disturbances and inflammatory pathways in depressive disorders [12, 13].

Notably, inflammation and insulin resistance do not operate independently but exhibit bidirectional, synergistic interactions in depression pathogenesis. Inflammatory cytokines (IL‑1β, IL‑6 and TNF‑α) impair insulin signalling via activation of stress kinases such as IKK‑β and JNK, which promote serine phosphorylation of insulin receptor substrate‑1 and subsequent insulin resistance [14, 15, 16]. In the central nervous system, this vicious cycle disrupts glucose metabolism, impairs neurotransmitter synthesis (serotonin and dopamine), compromises blood–brain barrier integrity and activates microglia, collectively contributing to depressive symptomatology [17, 18, 19, 20]. This mechanistic crosstalk highlights the rationale for composite biomarkers that capture both pathways simultaneously [21].

The CRP–triglyceride glucose index (CTI) represents a novel approach for combining inflammatory and metabolic markers. Initially developed and validated in cancer disease research [22], CTI has demonstrated superior predictive value for adverse cardiometabolic outcomes compared with its individual components, with subsequent studies confirming its effectiveness in predicting cardiovascular–kidney–metabolic syndrome, type 2 diabetes progression and cardiovascular mortality across diverse populations [23, 24, 25]. Unlike traditional single biomarkers such as CRP or TyG index alone, CTI captures the synergistic interaction between chronic low-grade inflammation and insulin resistance, which are two pathophysiological mechanisms increasingly recognised as central to depression pathogenesis [26]. Although other composite biomarkers have been explored in depression research, including neutrophil-to-lymphocyte ratio, systemic immune-inflammation index and remnant cholesterol [27, 28, 29], CTI offers distinct advantages: it is derived from readily available, standardized clinical laboratory tests with low intra-individual variability and high reproducibility. Prior research has explored associations between separate components (CRP or TyG) and depressive disorders [4, 5, 30]. However, the potential utility of an integrated marker remains largely unexplored. CTI targets the pathophysiologic axis where low-grade inflammation and insulin resistance co-occur, which is central to multiple disease processes. Notably, CTI has demonstrated superior predictive performance compared with TyG or CRP alone in cardiovascular disease (CVD) contexts, showing elevated C-statistics, net reclassification improvement (NRI) and integrated discrimination improvement (IDI) when added to baseline models [31]. Furthermore, CTI comprehensively assesses both pathways simultaneously through routine fasting blood tests already performed in clinical practice [32]. However, despite these advantages in cardiometabolic prediction, the CTI–depression relationship remains unexplored. Therefore, this study examined the association between CTI and depressive symptoms in a nationally representative sample. Given the established bidirectional relationship between inflammation and insulin resistance [33, 34], evaluating CTI in relation to depressive symptomatology may advance knowledge regarding depression’s mechanistic foundations and facilitate improved identification of at-risk populations.

## Material and Methods

### Study Population and Design

We conducted a cross-sectional analysis utilizing National Health and Nutrition Examination Survey (NHANES) data from 2005 to 2023. The NHANES programme, managed by the NCHS, represents a nationally representative initiative evaluating health and nutrition parameters among the US non-institutionalized civilian population. The survey employed a complex, multistage probability sampling design and included comprehensive health examinations, laboratory tests and in-person interviews conducted at mobile examination centres (MECs).

**Fig. 1.**
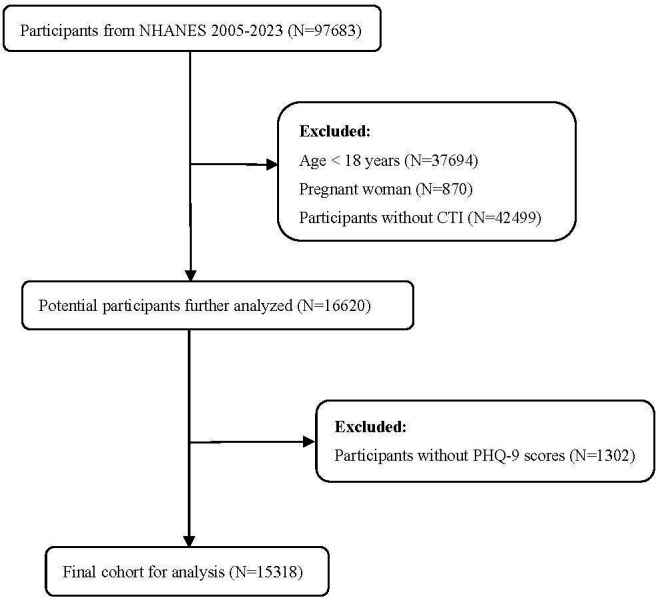
**Flowchart depicting the participant selection process from NHANES 2005–2023**. The diagram illustrates the sequential selection process of study participants from NHANES 2005–2023. NHANES, National Health and Nutrition Examination Survey.

Of the initial 97,683 participants across ten 2-year cycles (2005–2023), we applied the following exclusion criteria: (1) participants aged <18 years (n = 37,694); (2) pregnant women (n = 870); and (3) participants without complete data for CTI calculation (n = 42,499), resulting in 16,620 potential participants for further analysis. After excluding participants without Patient Health Questionnaire-9 (PHQ-9) scores (n = 1302), a final analytic sample of 15,318 individuals was included in the study (Fig. 1).

### Depression Assessment

Depressive symptomatology was evaluated through PHQ-9, a validated instrument measuring depression severity during the preceding two-week period [35]. This nine-item questionnaire aligns with DSM-IV diagnostic standards for depression [36], utilizing a four-point Likert scale where responses range from 0 (not at all) to 3 (nearly every day). Cumulative scores range from 0 to 27, with elevated values reflecting high symptom severity. Within our study protocol, participants responded to PHQ-9 at mobile examination centres via a computer-assisted personal interviewing platform under guidance from trained personnel. We applied a threshold score of ≥10 to denote clinically significant depressive symptoms. Prior validation research has established this cut-off point’s diagnostic accuracy at 88% for sensitivity and specificity in detecting major depression [35, 36, 37]. PHQ-9 demonstrates excellent internal reliability (Cronbach’s α = 0.89) and test–retest reliability (r = 0.84) [35]. The instrument showed strong construct validity, correlating highly with mental health measures (r = 0.73) and discriminating between levels of functional impairment and healthcare utilization, supporting its use in epidemiological research.

### Measurement of the CTI

The CTI was calculated using measurements obtained from blood samples collected after a minimum 8.5-hour fasting period. Laboratory analyses were performed in NCHS-certified facilities. We calculated CTI according to the equation: CTI = 0.412 × Ln(CRP) + TyG [22], wherein TyG denotes the triglyceride–glucose index, derived as Ln[fasting triglyceride (mg/dL) × fasting plasma glucose (mg/dL)/2] [10]. Laboratory measurements were conducted using standardized procedures. Serum triglyceride levels were quantified using enzymatic assays on the Roche Modular P and Roche Cobas 6000 chemistry analysers (Roche, Basel, Switzerland). FPG concentrations were determined via the oxygen rate methodology utilizing a Beckman DxC800 analyser (Roche, Basel, Switzerland). C-reactive protein (CRP) levels were determined through latex-enhanced nephelometry using a Behring Nephelometer (Siemens Healthineers, Erlangen, Germany). High CTI values indicate severe inflammation and insulin resistance. Detailed information regarding laboratory procedures and quality control measures are open at the NHANES official website (https://wwwn.cdc.gov/nchs/nhanes/Default.aspx).

### Measurement of Covariates

Our study incorporated various confounding factors requiring statistical control. Demographic parameters included age stratification (<60, ≥60 years), biological sex (male/female), race/ethnicity classification (Mexican American, Non-Hispanic White, Non-Hispanic Black and other), educational achievement (sub-high school, high school equivalent and post-high school) and partnership status (married/not married). Socioeconomic position was indexed using the Family Poverty Income Ratio, which compares household income with family size-specific poverty guidelines. BMI calculation followed standard methodology: weight (kg)/height^2^ (m^2^).

Clinical comorbidities were evaluated through participant self-reports combined with measured parameters. The definition of CVD encompassed self-reported heart failure, coronary artery disease, angina pectoris, myocardial infarction or stroke. Diabetes diagnosis was determined through self-reports, antidiabetic medication usage or HbA1c ≥6.5%. Hypertension diagnosis incorporated self-reports, blood pressure medication, systolic readings ≥130 mmHg or diastolic readings ≥80 mmHg [38]. Presence of malignancy was derived from self-reports. Pharmacological treatment data captured antidepressant and statin use (yes/no categories). Laboratory biomarkers, namely, creatinine, uric acid, AST, ALT and LDL-C, were quantified via standardised methodologies at NCHS-accredited laboratories.

### Statistical Analyses

Our analytical approach accounted for NHANES’ multistage probability sampling framework and survey weights. Specifically, we utilised the NHANES-provided examination sample weights (WTMEC2YR for single cycles) to generate nationally representative estimates. For multi-cycle analyses spanning 2005–2023, we created new weights by dividing the 2-year weights by the number of cycles (n = 10), as recommended by NCHS guidelines. All analyses incorporated the appropriate strata and primary sampling unit variables to account for the complex survey design, with variance estimates calculated using Taylor series linearization methods. Descriptive statistics included mean ± standard deviation for continuous variables following normal distribution, median (interquartile range) for non-normally distributed continuous data and count (percentage) for categorical measures. Normality of continuous variables was assessed using the Shapiro–Wilk test and visual inspection of Q-Q plots. Between-tertile comparisons employed ANOVA for normally distributed continuous variables, Kruskal–Wallis tests for skewed continuous data and chi-square analysis for categorical variables. Multivariable logistic regression evaluated CTI–depression associations through sequential modelling: Model I (unadjusted); Model II (incorporating sex, age, BMI, race/ethnicity, educational attainment, marital status and Family Poverty Income Ratio); and Model III (additionally controlling for diabetes, hypertension, CVD, creatinine, uric acid, LDL-C, AST, malignancy, statin therapy and antidepressant use). Findings are presented as ORs with corresponding 95% CIs. Restricted cubic spline methodology assessed potential nonlinearity in the CTI–depression relationship, applying full Model III covariate adjustment. Nonlinearity evaluation utilised likelihood ratio testing, comparing models with linear terms only versus those incorporating cubic spline components. Subgroup examinations explored CTI–depression associations across strata defined by sex, age (<60 vs ≥60 years), race/ethnicity, education, marital status and comorbidity burden. Effect modification was evaluated via likelihood ratio tests comparing nested logistic regression models with and without interaction terms for CTI and the stratification variable. Specifically, we compared models containing only main effects versus models including the multiplicative interaction term, with *p*-values derived from chi-square tests indicating whether the CTI–depression association differed significantly across subgroups. Statistical computations utilized R software (v4.0.0, R Foundation for Statistical Computing, Vienna, Austria) and EmpowerStats (X&Y Solutions, Inc., Boston, MA, USA). Statistical significance was defined as two-sided *p* < 0.05.

## Results

### Baseline Characteristics of Study Participants

A total of 15,318 individuals were included in the analysis. The median CTI was 7.85 (interquartile range: 6.78–9.15), with a range of 3.24–14.67. Participants were stratified into tertiles based on their CTI levels: low tertile (CTI <7.12, n = 5106), middle tertile (CTI 7.12–8.74, n = 5106) and high tertile (CTI >8.74, n = 5106). Significant differences in baseline characteristics were observed across CTI tertiles (Table 1). Individuals in the uppermost CTI tertile demonstrated greater age (*p* < 0.001) and elevated BMI (*p* < 0.001) compared with those in the lowermost tertile. Laboratory parameters, including uric acid, LDL cholesterol and liver enzymes (AST and ALT), showed progressive increases across CTI tertiles (all *p* < 0.001).

**Table 1.** **Baseline participant features according to CTI tertile classification**.

Variables		Low (N = 5106)	Middle (N = 5106)	High (N = 5106)	*p*-value
Age, years		40.0 (26.0–58.0)	52.0 (35.0–66.0)	54.0 (41.0–66.0)	<0.001
BMI, kg/m^2^		24.6 (21.9–28.0)	28.6 (25.3–32.5)	31.6 (27.6–36.7)	<0.001
Family Poverty Income Ratio		2.4 (1.2–4.4)	2.2 (1.2–4.1)	1.9 (1.1–3.6)	<0.001
Creatinine, mg/dL		0.8 (0.7–1.0)	0.9 (0.7–1.0)	0.8 (0.7–1.0)	<0.001
Uric acid, mg/dL		4.9 (4.1–5.8)	5.6 (4.6–6.4)	5.8 (4.9–6.8)	<0.001
AST, U/L		21.0 (18.0–25.0)	22.0 (18.0–26.0)	22.0 (18.0–28.0)	<0.001
LDL Cholesterol, mg/dL		98.0 (80.0–120.0)	113.0 (90.0–137.0)	115.0 (91.0–140.8)	<0.001
PHQ-9 score		2.0 (0.0–4.0)	2.0 (0.0–4.0)	2.0 (0.0–5.0)	<0.001
ALT, U/L		17.0 (14.0–23.0)	20.0 (16.0–28.0)	22.0 (16.0–31.0)	<0.001
Sex					<0.001
	Female	2623 (51.37%)	2423 (47.45%)	2579 (50.51%)	
	Male	2483 (48.63%)	2683 (52.55%)	2527 (49.49%)	
Race/Ethnicity					<0.001
	Mexican American	679 (13.30%)	849 (16.63%)	1037 (20.31%)	
	Non-Hispanic Black	1370 (26.83%)	1096 (21.46%)	835 (16.35%)	
	Non-Hispanic White	1981 (38.80%)	2091 (40.95%)	2218 (43.44%)	
	Other	1076 (21.07%)	1070 (20.96%)	1016 (19.90%)	
Education level					<0.001
	Below high school	824 (17.99%)	1155 (23.61%)	1479 (29.50%)	
	High school graduate	1002 (21.88%)	1194 (24.41%)	1241 (24.75%)	
	Above high school	2754 (60.13%)	2543 (51.98%)	2294 (45.75%)	
Marital status					<0.001
	Unmarried	1513 (43.89%)	1473 (39.44%)	1519 (38.76%)	
	Married	1934 (56.11%)	2262 (60.56%)	2400 (61.24%)	
Diabetes					<0.001
	No	4812 (94.24%)	4387 (85.92%)	3459 (67.74%)	
	Yes	294 (5.76%)	719 (14.08%)	1647 (32.26%)	
Hypertension					<0.001
	No	3727 (72.99%)	2867 (56.15%)	2266 (44.38%)	
	Yes	1379 (27.01%)	2239 (43.85%)	2840 (55.62%)	
CVD					<0.001
	No	4243 (92.62%)	4293 (87.72%)	4221 (84.08%)	
	Yes	338 (7.38%)	601 (12.28%)	799 (15.92%)	
Malignancy					<0.001
	No	4214 (92.03%)	4419 (90.31%)	4402 (87.83%)	
	Yes	365 (7.97%)	474 (9.69%)	610 (12.17%)	
Antidepressants					<0.001
	No	4738 (92.83%)	4557 (89.30%)	4328 (84.88%)	
	Yes	366 (7.17%)	546 (10.70%)	771 (15.12%)	
Statins use					<0.001
	No	4457 (87.32%)	4037 (79.11%)	3838 (75.27%)	
	Yes	647 (12.68%)	1066 (20.89%)	1261 (24.73%)	
Depression					<0.001
	No	4740 (92.83%)	4714 (92.32%)	4527 (88.66%)	
	Yes	366 (7.17%)	392 (7.68%)	579 (11.34%)	

Note: Data are presented as mean ± standard deviation for continuous variables with normal distribution, median (interquartile range) for skewed continuous variables and count (percentage) for categorical variables. Percentages may not total 100% due to missing data for some categorical variables. CTI, C-reactive protein–triglyceride glucose index; BMI, body mass index; AST, aspartate transaminase; LDL, low density lipoprotein; PHQ-9, Patient Health Questionnaire-9; ALT, alanine aminotransferase; CVD, cardiovascular disease.

Notable demographic variations were observed, with the highest CTI tertile showing a reduced proportion of participants with above high school education (45.75% vs 60.13% in the lowest tertile, *p* < 0.001). Higher CTI tertiles exhibited significantly greater frequencies of chronic conditions, including diabetes (32.26% vs 5.76%), hypertension (55.62% vs 27.01%) and CVD (15.92% vs 7.38%) (all *p* < 0.001). Notably, the prevalence of depression demonstrated a consistent upward trend across CTI tertiles, from 7.17% in the lowest tertile to 11.34% in the highest tertile (*p* < 0.001). Additionally, the highest CTI tertile exhibited increased antidepressant usage rates compared with the lowest tertile (15.12% vs 7.17%, *p* < 0.001).

### Associations Between CTI and Depression

**Fig. 2.**
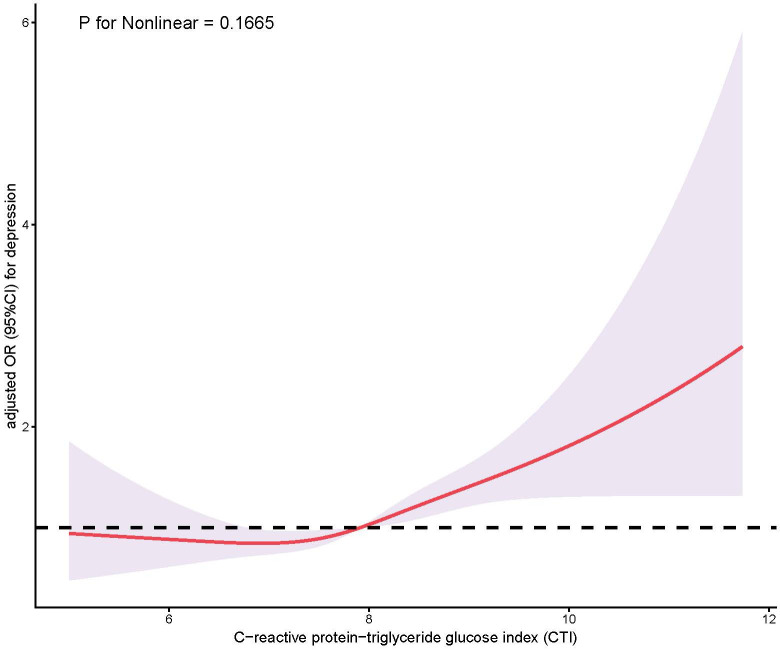
**Association between CTI and depressive symptom risk demonstrating linearity**. This graphic displays the CTI–depression dose–response pattern generated using restricted cubic spline methodology with knots positioned at the 10th, 50th and 90th percentiles. Adjusted ORs appear as a solid red line, with corresponding 95% CIs represented by the shaded area. The horizontal dashed line indicates OR = 1.0 (no association). ORs were adjusted for sex, age, BMI, CVD, race/ethnicity, creatinine, education level, malignancy, statins, marital status, uric acid, LDL cholesterol, family poverty income ratio, diabetes, hypertension, AST and antidepressant use. No significant nonlinear relationship was observed (*p* for nonlinearity = 0.1665). The Y-axis is plotted on a logarithmic scale.

Restricted cubic spline modelling confirmed a linear dose–response pattern between CTI and depression (*p* for nonlinearity = 0.1665), indicating that the risk of depression increased proportionally with CTI levels (Fig. 2).

We evaluated the CTI–depression association through continuous and categorical approaches with sequential covariate adjustment (Table 2). Continuous treatment with CTI indicated that each unit increment corresponded to a 31% heightened likelihood of depressive symptoms in crude analysis (OR = 1.31, 95% CI: 1.23–1.38, *p* < 0.0001). Following comprehensive adjustment for sociodemographic characteristics, lifestyle factors and clinical parameters, this relationship remained significant with modest attenuation (OR = 1.23, 95% CI: 1.11–1.36, *p* = 0.0001). Sensitivity analyses excluding participants on antidepressants or statins yielded consistent results, with CTI remaining significantly associated with depressive symptoms (fully adjusted OR: 1.29, 95% CI: 1.14–1.46, *p* < 0.0001; **Supplementary Table 1**).

**Table 2.** **Association of CTI with depressive symptoms, NHANES 2005–2023**.

Exposure		Model I OR (95% CI), *p*	Model II OR (95% CI), *p*	Model III OR (95% CI), *p*
CTI		1.31 (1.23, 1.38) <0.0001	1.27 (1.17, 1.39) <0.0001	1.23 (1.11, 1.36) 0.0001
CTI tertile				
	Low	1 (Reference)	1 (Reference)	1 (Reference)
	Middle	1.08 (0.93, 1.25) 0.3264	1.12 (0.91, 1.37) 0.2894	1.12 (0.90, 1.39) 0.3250
	High	1.66 (1.44, 1.90) <0.0001	1.54 (1.25, 1.90) <0.0001	1.48 (1.17, 1.86) 0.0009
*p* for trend		<0.0001	<0.0001	0.0005

Model I adjusted for: None; Model II adjusted for: sex, age, BMI, race/ethnicity, education level, marital status and family poverty income ratio; Model III adjusted for: sex, age, BMI, race/ethnicity, education level, marital status, family poverty income ratio, diabetes, hypertension, CVD, creatinine, uric acid, LDL cholesterol, AST, malignancy, statins and antidepressant.

Analysing PHQ-9 scores as a continuous variable revealed that elevated CTI values were significantly correlated with increased depressive symptom burden (Table 3).

**Table 3.** **Association between CTI and PHQ-9 scores as a continuous outcome**.

Exposure		Model I β (95% CI), *p*	Model II β (95% CI), *p*	Model III β (95% CI), *p*
CTI		0.44 (0.37, 0.51) <0.0001	0.36 (0.27, 0.46) <0.0001	0.29 (0.18, 0.40) <0.0001
	Low	0	0	0
	Middle	0.14 (−0.02, 0.30) 0.0939	0.11 (−0.10, 0.31) 0.3190	0.11 (−0.09, 0.31) 0.2948
	High	0.91 (0.75, 1.08) <0.0001	0.63 (0.41, 0.85) <0.0001	0.50 (0.27, 0.73) <0.0001
*p* for trend		<0.0001	<0.0001	<0.0001

Model I adjusted for: None; Model II adjusted for: sex, age, race/ethnicity, education level, marital status, family poverty income ratio and BMI; Model III adjusted for: sex, age, BMI, race/ethnicity, education level, marital status, family poverty income ratio, diabetes, hypertension, CVD, creatinine, uric acid, LDL cholesterol, AST, malignancy, statins and antidepressant.

Tertile-based categorisation of CTI revealed graded associations. Participants in the uppermost tertile exhibited markedly elevated depression risk versus those in the lowermost tertile (OR = 1.48, 95% CI: 1.17–1.86, *p* = 0.0009) following full confounder adjustment. The middle tertile demonstrated an elevated but non-significant risk (OR = 1.12, 95% CI: 0.90–1.39, *p* = 0.3250). A statistically significant linear gradient emerged across tertiles (*p* for trend = 0.0005), confirming a stepwise relationship between CTI magnitude and depressive symptomatology.

### Subgroup Analysis

**Fig. 3.**
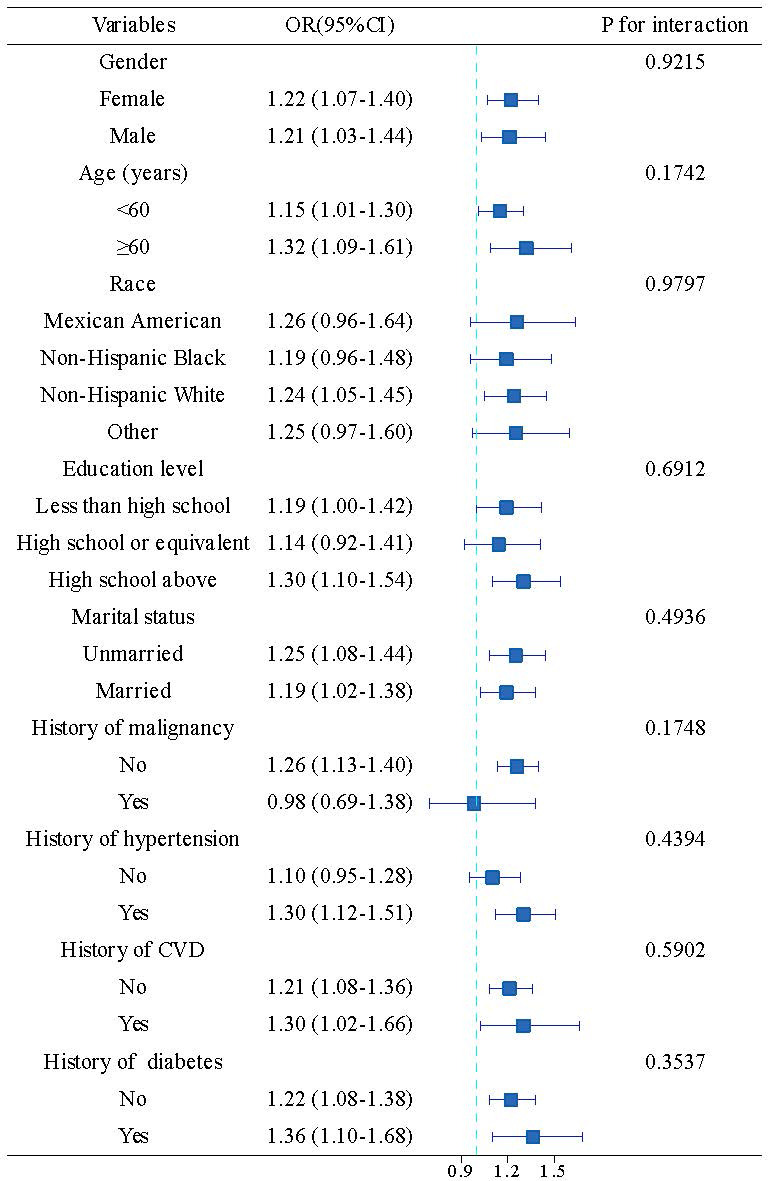
**Subgroup analyses of the association between CTI and depressive symptoms**. Forest plot showing odds ratios (ORs) and 95% confidence intervals (CIs) for the association between CTI (highest vs lowest tertile) and depressive symptoms across different subgroups. The vertical dashed line represents OR = 1 (no association). Squares represent point estimates, with horizontal lines indicating 95% CIs. *p* values for interaction tests between CTI and subgroup variables are shown on the right. The associations remained generally consistent across demographic and clinical characteristics, with no significant interactions observed (all *p* for interaction >0.05). The associations remained generally consistent across demographic and clinical characteristics, with no significant interactions observed (all *p* for interaction > 0.05). Statistically significant associations were observed in multiple subgroups, including participants aged ≥60 years (OR = 1.32, 95% CI: 1.09–1.61), those with above high school education (OR = 1.30, 95% CI: 1.10–1.54) and non-Hispanic White participants (OR = 1.24, 95% CI: 1.05–1.45). CVD, cardiovascular disease.

Stratified analyses were conducted to evaluate the consistency of the association between CTI and depressive symptoms across various subgroups (Fig. 3). The association remained statistically significant in multiple subgroups, including participants aged ≥60 years (OR = 1.32, 95% CI: 1.09–1.61), non-Hispanic White participants (OR = 1.24, 95% CI: 1.05–1.45) and those with above high school education (OR = 1.30, 95% CI: 1.10–1.54). Among participants stratified by malignancy history, the association was significant in those without malignancy (OR = 1.26, 95% CI: 1.13–1.40) but not in those with malignancy (OR = 0.98, 95% CI: 0.69–1.38). Furthermore, the relationship between CTI and depressive symptoms remained robust regardless of comorbidity status, with similar effect sizes observed in participants without diabetes (OR = 1.22, 95% CI: 1.08–1.38), hypertension (OR = 1.10, 95% CI: 0.95–1.28) or CVD (OR = 1.21, 95% CI: 1.08–1.36). The absence of significant interaction effects (all *p* for interaction >0.05) suggested that the association between CTI and depressive symptoms is relatively universal across different population subgroups, highlighting its potential as a broadly applicable biomarker for depression risk.

## Discussion

In this large, nationally representative study of US adults, we found a significant association between elevated CTI levels and increased odds of depressive symptoms. Several key findings emerged from our analysis. Firstly, participants in the highest CTI tertile demonstrated a 48% higher odds of depressive symptom compared with those in the lowest tertile, even after comprehensive adjustment for potential confounders. Secondly, our restricted cubic spline analysis revealed a linear relationship between CTI and depressive symptoms, characterized by a consistent and gradual increase in risk across the entire range of CTI values. Thirdly, the association remained robust across multiple adjustment models, suggesting the independence of this relationship from various demographic, lifestyle and clinical factors. Finally, the dose–response relationship observed across CTI tertiles, with a significant linear trend, strengthening the evidence for a meaningful association between CTI and depressive symptoms.

The relationship between CTI and depressive symptoms can be explained through several potential biological mechanisms. Firstly, elevated CRP levels indicate systemic inflammation, which has been shown to affect neurotransmitter systems and promote neuroinflammation [39, 40]. Our discovery that the highest CTI tertile showed significantly elevated depression prevalence and PHQ-9 scores suggested that this combined inflammatory–metabolic burden has clinical relevance beyond isolated pathway dysfunction. This inflammatory state can disrupt neurotransmitter metabolism and neural circuits involved in mood regulation [41]. Secondly, insulin resistance, reflected in the triglyceride–glucose component of CTI, may influence brain glucose metabolism and neurotransmitter function [42]. Previous research has demonstrated that insulin resistance can lead to alterations in brain structure and function, potentially contributing to depressive symptoms [43]. The linear dose–response relationship supports a continuous biological gradient consistent with mechanistic pathways. Furthermore, persistent CTI–depression associations in participants with and without metabolic comorbidities suggest that CTI captures subclinical dysfunction relevant to depression even before the emergence of overt disease. Although our cross-sectional design prevents causal inference, the observed dose–response relationship, consistency across subgroups and correlation with symptom severity provide empirical patterns consistent with these mechanistic hypotheses. Furthermore, elevated triglycerides may affect blood–brain barrier function, potentially influencing the central nervous system’s inflammatory state and neurotransmitter systems [44].

Our subgroup analyses revealed several important patterns. The consistent association observed across age groups, including adults aged ≥60 years (OR = 1.32, 95% CI: 1.09–1.61), was aligned with previous findings suggesting age-related differences in inflammatory responses [45, 46]. The association among those with less than high school education (OR = 1.19, 95% CI: 1.00–1.42) may reflect the complex interplay among socioeconomic status, metabolic health and mental well-being. Racial/ethnic variations in the CTI–depression relationship, with associations among non-Hispanic Whites (OR = 1.24, 95% CI: 1.05–1.45), suggest potential genetic or environmental influences on this relationship. Notably, the consistency of findings across comorbidity status indicates that the CTI–depression association is independent of common metabolic conditions.

Our findings were generally consistent with but also extended beyond those reported by Huang *et al*. [47] in their analysis of CTI and depressive symptoms using NHANES data. Both studies demonstrated a significant positive association between elevated CTI levels and increased risk of depressive symptoms in US adults. However, several key differences exist between the two investigations. Our study examined an extended time period with an expanded sample size. Additionally, consistent with Huang *et al*. [47], our restricted cubic spline analysis confirmed a linear relationship between CTI and depressive symptoms (*p* for nonlinearity = 0.1665), with a consistent increase in risk across the entire range of CTI values. Compared with the prior NHANES study by Huang *et al*. [47] covering 2005–2010, our investigation extended the analysis to 2005–2023 with a 2.6-fold larger sample (N = 15,318 vs 5954), incorporating additional critical covariates and demonstrating the temporal stability of the CTI–depression association across changing population characteristics. This extended temporal validation strengthens the clinical relevance of CTI as a screening tool in contemporary practice.

These findings have several important clinical implications. Firstly, CTI could serve as a valuable screening tool for identifying individuals at increased risk of depression, particularly given its integration of readily available clinical markers [41]. The nonlinear relationship suggests that even slight increases in CTI may warrant attention in clinical settings. Secondly, our results support the potential value of targeting metabolic health in depression prevention strategies. The strong associations observed in certain subgroups, particularly older adults and those with lower educational levels, indicate populations that may derive the greatest advantage from targeted screening and intervention. Thirdly, the consistency of findings across comorbidity status suggests that CTI may be a useful marker regardless of underlying metabolic conditions.

Our study has several notable strengths. The large, nationally representative sample enhances the generalizability of our findings to the US adult population. The comprehensive adjustment for potential confounders, including sociodemographic factors, lifestyle behaviours and clinical covariates, strengthens the validity of our results [48]. The novel integration of inflammatory and metabolic markers through CTI provides a more comprehensive assessment of metabolic dysfunction than individual markers alone. Additionally, our robust statistical methodology, including restricted cubic spline analysis, facilitated a comprehensive characterisation of the relationship between CTI and depressive symptoms.

These findings have important clinical implications accompanied with specific practical recommendations. Firstly, CTI could be integrated into existing cardiovascular risk assessment as an opportunistic depression screening tool. Based on our tertile analysis, individuals with CTI >8.74 (highest tertile, OR = 1.48) warrant routine PHQ-9 screening, whereas those with CTI 7.12–8.74 may benefit from monitoring. Notably, CTI requires no additional costs beyond standard fasting lipid panels and CRP currently recommended for cardiovascular assessment. Secondly, our subgroup findings identified priority populations: older adults (≥60 years, OR = 1.32) and individuals with low educational attainment (OR = 1.30) would derive the greatest benefit from integrated cardiometabolic–mental health interventions. Specific evidence-based interventions include Mediterranean diet, structured exercise programmes (≥150 min weekly) and collaborative care models. Thirdly, for patients with elevated CTI, treatment optimisation should prioritise agents with anti-inflammatory properties (e.g., metformin) or mood benefits (e.g., GLP-1 agonists), enabling the integrated management of metabolic and mental health.

Several limitations should be considered when interpreting our findings. Firstly, the cross-sectional design prevents causal inference regarding the relationship between CTI and depressive symptoms [49]. Moreover, reverse causality cannot be excluded; depression may elevate inflammatory markers and worsen metabolic dysfunction through behavioural changes (reduced physical activity and poor dietary choices), HPA axis dysregulation and autonomic nervous system alterations, making the directionality of associations uncertain. Longitudinal studies are needed to disentangle temporal relationships. Secondly, although we adjusted for numerous confounders, residual confounding from unmeasured factors cannot be ruled out. Thirdly, the single-time-point measurements of CTI and depressive symptoms may not capture the dynamic nature of these parameters over time [50]. Fourthly, PHQ-9 is a validated screening tool for depressive symptoms, but it relies on self-reports, which may include recall bias or social desirability bias. Nonetheless, its robust psychometric properties and widespread validation support its use in population-based research. Additionally, PHQ-9 assesses symptom severity rather than clinical diagnosis, potentially including individuals with subsyndromal symptoms. The absence of medication data represents a critical unmeasured confounder. Antidepressants, anti-inflammatory drugs and statins may independently alter CTI or depression, thereby skewing observed associations in either direction. Future longitudinal studies with medication documentation and serial CTI measurements are essential to establish temporal sequences and causal relationships.

## Conclusion

Our study demonstrated a significant association between elevated CTI levels and increased odds of depressive symptoms in US adults, characterized by a linear dose–response relationship. These findings suggest that CTI, which combines inflammatory and metabolic markers, may serve as a valuable tool for identifying individuals at increased risk of depression. The strong associations observed in certain subgroups highlight populations that might benefit most from targeted screening and intervention strategies. Future longitudinal studies are needed to establish causality and evaluate the potential applicability of CTI in clinical practice. Our results highlight the importance of considering metabolic health in mental well-being and suggest new avenues for the prevention and management of depression.

## Availability of Data and Materials

The data used in this study are publicly available from the National Health and Nutrition Examination Survey (NHANES) database.
