# Supplementary material for: Association Between C-Reactive Protein–Triglyceride Glucose Index and Depressive Symptoms Among US Adults: A Nationally Representative Cross-Sectional Study From 2005 to 2023
Source: Actas Esp Psiquiatr. 2026 Apr 15;54(2):455–66. doi: 10.62641/aep.v54i2.2163 (PMC13180666; doi:10.62641/aep.v54i2.2163)
Supplement: Supplementary file 1 [file ActEsp-54-2-455-466-s1.zip › Supplementary Table 1.docx]

**Supplementary Table 1.** Sensitivity analyses of the association of CTI with depressive symptoms excluding participants taking antidepressants or statins

| Exposure | Model I OR (95%CI), *P* | Model II OR (95%CI), *P* | Model III OR (95%CI), *P* |
| --- | --- | --- | --- |
| CTI | 1.22 (1.14, 1.31) <0.0001 | 1.28 (1.15, 1.42) <0.0001 | 1.29 (1.14, 1.46) <0.0001 |
| CTI tertile |  |  |  |
| Low | 1 | 1 | 1 |
| Middle | 0.99 (0.83, 1.18) <0.0001 | 1.12 (0.87, 1.431) 0.3754 | 1.17 (0.91, 1.51) 0.2111 |
| High | 1.47 (1.24, 1.73) <0.0001 | 1.56 (1.21, 2.00) 0.0006 | 1.60 (1.22, 2.09) 0.0006 |
| *P* for trend | <0.0001 | 0.0003 | 0.00058 |

Model I adjust for: None

Model II adjust for: sex, age, BMI, race/ethnicity, education level, marital status, family poverty income ratio, and BMI;

Model III adjust for: sex, age, BMI, race/ethnicity, education level, marital status, family poverty income ratio, BMI, creatinine, uric acid, LDL Cholesterol, AST, malignancy.
